# Normalization of gene expression data revisited: the three viewpoints of the transcriptome in human skeletal muscle undergoing load-induced hypertrophy and why they matter

**DOI:** 10.1186/s12859-022-04791-y

**Published:** 2022-06-18

**Authors:** Yusuf Khan, Daniel Hammarström, Stian Ellefsen, Rafi Ahmad

**Affiliations:** 1 Department of Biotechnology, Inland Norway University of Applied Sciences, Holsetgata 22, 2317 Hamar, Norway; 2 Section for Health and Exercise Physiology, Department of Public Health and Sport Sciences, Inland Norway University of Applied Sciences, Lillehammer, Norway; 3 Innlandet Hospital Trust, Lillehammer, Norway; 4 Institute of Clinical Medicine, Faculty of Health Sciences, UiT - The Arctic University of Norway, Hansine Hansens veg 18, 9019 Tromsö, Norway

**Keywords:** RNA-seq, Skeletal muscle, Normalization, Resistance training

## Abstract

**Background:**

The biological relevance and accuracy of gene expression data depend on the adequacy of data normalization. This is both due to its role in resolving and accounting for technical variation and errors, and its defining role in shaping the viewpoint of biological interpretations. Still, the choice of the normalization method is often not explicitly motivated although this choice may be particularly decisive for conclusions in studies involving pronounced cellular plasticity. In this study, we highlight the consequences of using three fundamentally different modes of normalization for interpreting RNA-seq data from human skeletal muscle undergoing exercise-training-induced growth. Briefly, 25 participants conducted 12 weeks of high-load resistance training. Muscle biopsy specimens were sampled from m. vastus lateralis before, after two weeks of training (week 2) and after the intervention (week 12), and were subsequently analyzed using RNA-seq. Transcript counts were modeled as (1) per-library-size, (2) per-total-RNA, and (3) per-sample-size (per-mg-tissue).

**Result:**

Initially, the three modes of transcript modeling led to the identification of three unique sets of stable genes, which displayed differential expression profiles. Specifically, genes showing stable expression across samples in the per-library-size dataset displayed training-associated increases in per-total-RNA and per-sample-size datasets. These gene sets were then used for normalization of the entire dataset, providing transcript abundance estimates corresponding to each of the three biological viewpoints (i.e., per-library-size, per-total-RNA, and per-sample-size). The different normalization modes led to different conclusions, measured as training-associated changes in transcript expression. Briefly, for 27% and 20% of the transcripts, training was associated with changes in expression in per-total-RNA and per-sample-size scenarios, but not in the per-library-size scenario. At week 2, this led to opposite conclusions for 4% of the transcripts between per-library-size and per-sample-size datasets (↑ vs. ↓, respectively).

**Conclusion:**

Scientists should be explicit with their choice of normalization strategies and should interpret the results of gene expression analyses with caution. This is particularly important for data sets involving a limited number of genes or involving growing or differentiating cellular models, where the risk of biased conclusions is pronounced.

## Introduction

In gene expression analyses, data normalization can be performed using a multitude of approaches, acting as a significant determinant of the validity and reliability of interpretations [1–4]. For any data set, available normalization strategies are, at least partially, predetermined by the technique used for data acquisition. Still, normalization always involves a myriad of explicit choices that may profoundly affect analytical outcomes. For example, for studies utilizing quantitative PCR (qPCR), the selection of internal reference genes will largely define downstream analyses and conclusions, and the utilization of non-validated reference genes will lead to substantial bias [4]. Analogous to this, for studies involving RNA sequencing, appropriate library size scaling will determine the comparability of samples in downstream statistical analysis [1, 3]. Overall, data normalization essentially targets sources of technique-specific artifacts and non-biological variation. In addition, it also defines the biological perspective from which data are interpreted [2, 5]. This means that the choice of normalization mode will define the biological output of the experiment. Indeed, transcript abundances can be modeled using either of three distinctly different approaches; abundances relative to the overall mRNA pool (1; i.e., using geometric averaging; per-library-size), abundances relative to the total amount of RNA (2; per-total-RNA), or abundances relative to amounts of tissue or numbers of cells used in the experiment (3; per-sample-size) [2, 5].

While each of these perspectives holds biological merit, providing gene expression data that can be interpreted and compared between samples (e.g., changes from before to after a specific treatment), they do so in different manners. First, the per-library-size approach provides data that assess the relative abundances of transcripts relative to all other transcripts, arguably enabling assessment of transcript expression that compares their ability to compete for slots on ribosomes. Second, the per-total-RNA approach provides data that assess the absolute level of transcripts relative to the entire pool of RNA, enabling assessment of transcript expression that compares their ability to recognize and bind to ribosomes. Third, the per-sample-size approach provides data that assess the overall abundances of transcripts in the biological sample at hand, and thus their content per-cell or per-tissue weight. Consequently, the three different normalization scenarios set the stage for interpretations with different biological perspectives. These differences will be exacerbated in experimental models and designs involving large degrees of cellular perturbations and plasticity, with accompanying changes in the overall mRNA and total RNA expression [5–7]. Despite this, the analytical consequence of using a specific normalization strategy is rarely explicitly addressed in the biomedical literature, even though it represents an old and ever-present issue in mRNA-based analyses [2, 5].

The present study aimed to investigate the consequences of using each of three normalization modes (per-library-size, per-total-RNA, and per-sample-size) for transcriptome profiling of RNA-seq data from a highly plastic model of human biology. Briefly, twenty-five human participants conducted twelve weeks of high-load resistance training using a within-participant study design. Each participant performed exercise training with either low or moderate volume, allocated to either leg [8]. Overall, both study protocols led to substantial changes in muscle strength, mass, and phenotype. The latter was evaluated from bilateral muscle biopsies (m. vastus lateralis) sampled at baseline and after two and twelve weeks of training. Biopsy samples showed marked increases in overall total RNA and mRNA abundances, arguably making it an adequate experimental system for the proposed comparison [8, 9]. In the current analyses, the first objective was to identify a subset of gene transcripts that show relative stability within participants across all time points, measured as transcript abundances per-library-size, per-total-RNA, and per-sample-size, respectively. Secondly, we used the resulting reference gene sets to normalize the entire RNA-seq dataset, ultimately providing estimates of transcript abundances corresponding to each of the three perspectives of normalization.

## Methods

### Study overview

Thirty-four participants completed a 12-week progressive resistance training intervention with legs randomly allocated to either low (one set per exercise) or moderate-volume (three sets per exercise) training, as previously described [8]. The training intervention consisted of leg-press, leg-curl, and knee-extension. Bilateral muscle biopsies were obtained before the intervention and after two and twelve weeks of training. Total RNA was extracted from the biopsy material (TRIzol, ThermoFisher Scientific, Oslo, Norway) [8], and samples were selected for analysis based on RNA integrity scores. Twenty-five participants had a complete set of samples with integrity scores $\ge$ 7 (Average RQI 9, SD: 0.4; Experion Automated Electrophoresis Station using RNA StdSens Assay, Bio-Rad, Norway) and were selected for the RNA-sequencing experiment [9]. A fixed amount of total RNA (1000 ng) was depleted of ribosomal RNA and used for RNA-seq library preparations and subjected to Paired-end sequencing (Illumina HiSeq 3000, Illumina, San Diego, CA USA), as detailed elsewhere [9]. For the present analyses, data from the two legs/volume conditions were treated as biological replicates during data modeling, with interpretations focusing on the effects of resistance training per se rather than on differential effects of the two training volume conditions.

### Preprocessing, read alignment, and quantification

Before alignment, Trimmomatic (version 0.39) [10] was used to filter out low-quality reads and remove poor-quality bases and adaptor sequences using default settings. The quality of filtered files was calculated using FastQC (version 0.11.4). After quality filtering, reads were aligned to the human genome and quantified on the level of transcripts using RSEM (version 1.3.1) [11] and GRCh38 release-97 (downloaded from http://ftp.ensemble.org/).

### Identification of stable genes and modeling of transcript counts

**Fig. 1:**
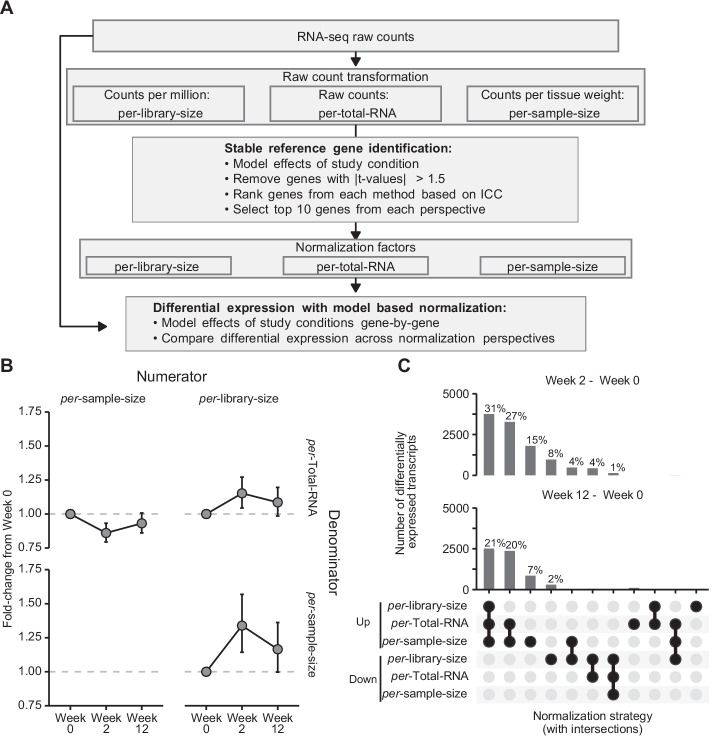
**A** Overview of data transformation and analyzes used. Raw counts were transformed to represent normalized data per-library-size, per-total-RNA and per-sample-size (tissue mass). Transformed counts were used to identify stable reference genes free from systematic effect and with subsequent ranking by intra-class correlation. Normalization factors comprised of 10 transcripts from each normalization approach was used in differential expression analysis. **B** Fold-changes of sample references (average of the top ten stable transcripts per normalization mode) ratios with numerators plotted over columns and denominators over rows. Error bars represent 95% CI. **C** Transcripts identified as differentially up and down-regulated over time (differences from Week 0 to Week 2 and 12 respectively) from generalized linear models with each normalization factor used as a model offset. Percentages represents proportions of all transcripts identified as differentially expressed regardless of normalization approach. Up- and down-regulation determined from false discovery rate-adjusted *p* values (*p* < 0.05). Black points represent intersections, e.g., where the same transcript has been identified from in one or more normalization perspective

The overall assumption of the analyses was that modeling of transcript expression to crude estimates of the three biological denominators mRNA, total RNA, and sample size would be formative for downstream analyses due to incomparable scaling and measurement errors between normalization modes, and thus would affect interpretations (Fig. 1A).

Initially, we, therefore, identified internal reference genes (stable genes) to create comparable normalization factors between normalization modes for subsequent analyses. Stable genes were selected from a subset of transcripts that showed robust expression across all samples, filtered with the minimum count per sample set to 30. After filtering, 5687 genes remained in the data set for assessment of within-participant stability. For each normalization mode (per-library-size, per-total-RNA and per-sample-size), stable genes were then assessed using transcript counts transformed to counts per million (CPM), calculated as counts per scaled library size (total counts scaled by trimmed mean of M-values [1]), counts per amount of tissue (mg of tissue $\times {10}^{6}$) and as counts per total RNA ($1\times {10}^{6}$, assuming equal total RNA in each reaction), respectively. CPM values were log-transformed before being fitted to linear mixed-effects models on a target-by-target basis. Models were subsequently used to (1) assess the effects of the intervention on transcript abundances over time and to (2) determine the intraclass correlation coefficient (ICC), defined as the amount of variance attributed to between-participant variation relative to the total variance. For each normalization mode, maximal t-values calculated from model coefficients representing study conditions (time and exercise volume) were used to remove transcripts showing indices of the intervention's systematic effects. Transcripts with absolute t-values < 1.5 were subjected to subsequent ranking based on ICC values. The top ten transcripts from each normalization mode were then defined as stable reference genes, which were deemed suitable for calculating normalization denominators. Selected transcripts were scaled (${x}_{1}/max(x)$) and averaged per sample to form the sample reference. To compare sample references from each normalization mode, ratios were evaluated over time from estimates obtained from linear mixed-effects models.

Thereafter, the complete set of transcripts (excluding reference genes, filtered with minimum count = 1, n = 12,066) was modeled on a target-by-target basis using negative binomial generalized linear mixed models (GLMM) [9, 12], with normalization mode-specific normalization factors being used as offsets in each model fit to express gene counts per-library-size, per-total-RNA and per-sample-size. Model fits were used to assess the effects of study conditions on relative gene counts. For the sake of this analysis, samples from each leg were considered biological replicates to determine the impact of exercise training per se (time-effects). Differentially expressed genes were defined as significantly different from baseline on a target-by-target basis (*p* < 0.05 adjusted for false discovery rate, FDR).

## Results

The training intervention led to robust increases in muscle mass and strength (on average 4% and 25%, respectively [8]). This was accompanied by an increase in total RNA from baseline to weeks two and twelve (on average 27% and 17%, respectively) and an increase in the sequenced library size, despite a lower amount of tissue being used during library preparations [8, 9]. In the present RNA-seq dataset, the initial modeling of data, providing gene expression estimates relative to per-library-size, per-total-RNA, and per-sample-size, led to the identification of three unique clusters of stable gene transcripts across untrained and resistance-trained muscle specimens. Each of these gene clusters consisted of genes that showed unaltered expression across muscle biopsies sampled from each participant (and as such was not affected by the resistance training). The number of genes in the three clusters varied substantially between modes of modeling (per-library-size, n = 1266; per-total-RNA, n = 90; per-sample-size, n = 18), with per-library-size normalization being associated with higher ICC estimates, suggesting higher degrees of consistency between samples. Based on ICC estimates, the top ten most stable transcripts from each modeling scenario were then identified (Table 1). While there was no overlap between the per-library-size cluster and either of the two other clusters among these transcripts, per-total-RNA and per-samples-size datasets contained two overlapping transcripts (Table 1).

**Table 1:** Genes selected as stable reference genes from each normalization scenario

Normalization strategy	Transcript ID	Gene symbol	Gene biotype	Intraclass correlation
Per-library-size	ENST00000643905			0.915
	ENST00000439211	DHFR	Protein coding	0.877
	ENST00000582787	SP2-DT	lncRNA	0.873
	ENST00000342992	TTN	Protein coding	0.866
	ENST00000361681	MT-ND6	Protein coding	0.864
	ENST00000371470	MAGOH	Protein coding	0.846
	ENST00000234256	SLC1A4	Protein coding	0.842
	ENST00000341162	FCF1	Protein coding	0.841
	ENST00000480046	METTL2B	Protein coding	0.839
	ENST00000295955	RPL9	Protein coding	0.828
Per-total-RNA	ENST00000445125		Processed pseudogene	0.715
	ENST00000312184	TMEM70	Protein coding	0.579
	ENST00000552002	CHURC1	Protein coding	0.559
	ENST00000357033	DMD	Protein coding	0.559
	ENST00000275300	SLC22A3	Protein coding	0.555
	ENST00000496823	BCL6	Protein coding	0.548
	ENST00000546248	TRDN	Protein coding	0.522
	ENST00000309881	CD36	Protein coding	0.505
	ENST00000005178	PDK4	Protein coding	0.496
	ENST00000522603	ASPH	Protein coding	0.492
Per-sample-size	ENST00000496823	BCL6	Protein coding	0.536
	ENST00000546248	TRDN	Protein coding	0.497
	ENST00000216019	DDX17	Protein coding	0.461
	ENST00000005178	PDK4	Protein coding	0.458
	ENST00000361915	AGL	Protein coding	0.421
	ENST00000418381			0.416
	ENST00000294724	AGL	Protein coding	0.405
	ENST00000366645	EXOC8	Protein coding	0.391
	ENST00000261349	LRP6	Protein coding	0.384
	ENST00000306270	IBTK	Protein coding	0.328

Based on the three clusters of top-ten stable genes, we then computed a scaled average of stable transcript expression for each mode of modeling. In these analyses, the stable transcripts identified in the per-library-size dataset showed clear training-associated increases in abundances in per-total-RNA and per-sample-size datasets (Fig. 1B, right panels). Similarly, stable genes identified in the per-sample-size dataset showed decreased expression in the per-total-RNA dataset (Fig. 1B, upper left panel). These differences were most pronounced in samples obtained after two weeks of resistance training (Fig. 1B).

We utilized the three clusters of stable transcripts for normalization of the entire RNA-seq dataset to identify training-induced differentially expressed transcripts per normalization mode on a transcript-by-transcript basis. Figure 1C illustrates the overlap of transcripts identified as differentially expressed (up and down-regulated from baseline) across normalization modes with black filled circles indicating which normalization modes are included in each set. Most transcripts identified as differentially up-regulated (31% and 21% of transcripts identified as differentially expressed from baseline to weeks 2 and 12, respectively) were commonly identified in all three normalization modes (Fig. 1C, first column). In contrast, 27% and 20% of the transcripts identified as differentially expressed, differential expression was only identified in per-total-RNA and per-sample-size scenarios, but not in the per-library-size scenario (Fig. 1C, second column). Furthermore, 15% and 7% of the genes identified as differentially expressed were uniquely identified as up-regulated using the per-sample-size normalization mode (Fig. 1C, third column) whereas most transcripts identified as uniquely down-regulated were found in the per-library-size normalization mode (Fig. 1C, fourth column). At week 2, discrepancies between normalization modes led to transcripts (4%) being identified as up-regulated in the per-sample-size mode but down-regulated in the per-library-size mode.

## Discussion

The present study demonstrates that the choice of normalization modality will affect the outcome of gene expression analyses in models of load-induced skeletal muscle plasticity in humans. The three modes of normalization (per-library-size, per-total-RNA, and per-sample size) were associated with different patterns of training-associated changes in gene expression. In general, per-library-size-based normalization was associated with the underestimation of mRNA abundances compared to the two other approaches. This underestimation was attributed to an overall increase in total RNA and mRNA expression in the muscle samples [8], with per-library-size-based analyses inherently assuming global transcript expression to remain unchanged across samples. Despite this, library size-based normalization remains the point of reference for most transcriptome studies [3, 13], which is also true for studies investigating responses to exercise training (e.g., metamex) [14], even though such treatments typically lead to global-scale changes in transcription [8, 15, 16]. These observations advocate that the choice of normalization mode must be carefully evaluated in any study involving gene expression analyses to ensure adequate biological interpretations [7]. Indeed, for experimental models involving large degrees of cellular perturbations and plasticity, and thus potentially global transcription amplification, per-library-normalization will lead to underestimation of transcript abundances [15].

While the results from the present study indicate the impact of using different normalization strategies, they do not inform us on the rights and wrongs of normalization choices. The correct use of a specific normalization strategy depends on the research questions and model systems under study. For example, in systems where relative transcript abundances are of primary interest, or in models where whole-transcriptome expression remains stable, it seems prudent to use per-library-size normalization [1, 3, 5]. This will provide data with the appropriate biological viewpoint, essentially informing about the competitive ability of transcripts to recognize and associate with ribosomes. In addition, per-library-size normalization arguably leads to more explicit correction of the technical variation occurring during sample preparations (e.g., during RNA extraction). However, as exemplified in the present study, for systems where these assumptions are not met and global changes occur for variables such as total RNA and global mRNA expression, it may lead to biased conclusions [5]. In such studies, absolute transcript abundances provide an alternative and perhaps more suitable outcome. Indeed, cellular growth is likely to be associated with dose-dependent changes in protein accretion and speculatively also transcript abundances, leaving per-total-RNA and per-samples-size normalization as the most beneficial approach for interpreting cellular characteristics.

In most gene expression studies, a limited number of mRNA species is investigated using methods such as qPCR, with normalization generally being performed using geometric averaging of a presumed set of stable genes [4, 17] In such studies, the necessity of complying with the logics of the present study is reinforced. Indeed, they typically involve statistical analyses that do not adjust for the presence of multiple observations, as is the case in RNA-seq experiments. This amplifies the likelihood of detecting differential gene expression patterns between different modes of normalization. For such analyses, particular care is thus needed during data normalization, reiterating the need for selecting stable genes that adequately represent the biological viewpoint of desire (i.e., per-library-size vs. per-total-RNA vs. per-sample-size). For datasets involving resistance-trained human skeletal muscle, the stable gene clusters identified in the current analyses pose as potential candidates. However, their representativeness and stability must be validated separately in any given study, as they are likely to be affected by tweaks in study variables such as treatment protocols (e.g., differences in the resistance training modalities) and the nature of the biological model (e.g., variation in participant age and disease status) [8, 9, 18].

## Conclusion

In the present study, we show that the choice of normalization modality (per-library-size vs. per-total-RNA vs. per-sample-size) affects interpretations of transcriptome responses in a human model of load-induced skeletal muscle plasticity. For any gene expression study, data normalization should be conducted and evaluated with care and intent, ensuring the stability and representativity of normalization denominators, and importantly, the biological viewpoint of outcome measures.

## Data Availability

The codes and the count data used in analysis during the current study are available from the corresponding author on request. The raw sequence data is not publicly available due to patient confidentiality.

## Abbreviations

RNA: Ribonucleic acid
GLMM: Generalized linear mixed models
ICC: Intraclass correlation coefficient
CPM: Counts per million

## References

[1] Anders S, Huber W (2010). Differential expression analysis for sequence count data. Genome Biol.

[2] Huggett J, Dheda K, Bustin S, Zumla A (2005). Real-time RT-PCR normalisation; strategies and considerations. Genes Immun.

[3] Robinson MD, Oshlack A (2010). A scaling normalization method for differential expression analysis of RNA-seq data. Genome Biol.

[4] Vandesompele J, De Preter K, Pattyn F, Poppe B, Van Roy N, De Paepe A, Speleman F (2002). Accurate normalization of real-time quantitative RT-PCR data by geometric averaging of multiple internal control genes. Genome Biol.

[5] Coate JE, Doyle JJ (2015). Variation in transcriptome size: are we getting the message?. Chromosoma.

[6] Hansen MC, Nielsen AK, Molin S, Hammer K, Kilstrup M (2001). Changes in rRNA levels during stress invalidates results from mRNA blotting: fluorescence in situ rRNA hybridization permits renormalization for estimation of cellular mRNA levels. J Bacteriol.

[7] Loven J, Orlando DA, Sigova AA, Lin CY, Rahl PB, Burge CB, Levens DL, Lee TI, Young RA (2012). Revisiting global gene expression analysis. Cell.

[8] Hammarstrom D, Ofsteng S, Koll L, Hanestadhaugen M, Hollan I, Apro W, Whist JE, Blomstrand E, Ronnestad BR, Ellefsen S (2020). Benefits of higher resistance-training volume are related to ribosome biogenesis. J Physiol.

[9] Khan Y, Hammarstrom D, Ronnestad BR, Ellefsen S, Ahmad R (2020). Increased biological relevance of transcriptome analyses in human skeletal muscle using a model-specific pipeline. BMC Bioinform.

[10] Bolger AM, Lohse M, Usadel B (2014). Trimmomatic: a flexible trimmer for Illumina sequence data. Bioinformatics.

[11] Li B, Dewey CN (2011). RSEM: accurate transcript quantification from RNA-Seq data with or without a reference genome. BMC Bioinform.

[12] Cui S, Ji T, Li J, Cheng J, Qiu J (2016). What if we ignore the random effects when analyzing RNA-seq data in a multifactor experiment. Stat Appl Genet Mol Biol.

[13] Love MI, Huber W, Anders S (2014). Moderated estimation of fold change and dispersion for RNA-seq data with DESeq2. Genome Biol.

[14] Pillon NJ, Gabriel BM, Dollet L, Smith JAB, Sardon Puig L, Botella J, Bishop DJ, Krook A, Zierath JR (2020). Transcriptomic profiling of skeletal muscle adaptations to exercise and inactivity. Nat Commun.

[15] Chaillou T, Malgoyre A, Banzet S, Chapot R, Koulmann N, Pugniere P, Beaudry M, Bigard X, Peinnequin A (2011). Pitfalls in target mRNA quantification for real-time quantitative RT-PCR in overload-induced skeletal muscle hypertrophy. Physiol Genom.

[16] Figueiredo VC, McCarthy JJ (2019). Regulation of ribosome biogenesis in skeletal muscle hypertrophy. Physiology.

[17] Andersen CL, Jensen JL, Orntoft TF (2004). Normalization of real-time quantitative reverse transcription-PCR data: a model-based variance estimation approach to identify genes suited for normalization, applied to bladder and colon cancer data sets. Cancer Res.

[18] Brook MS, Wilkinson DJ, Mitchell WK, Lund JN, Phillips BE, Szewczyk NJ, Greenhaff PL, Smith K, Atherton PJ (2016). Synchronous deficits in cumulative muscle protein synthesis and ribosomal biogenesis underlie age-related anabolic resistance to exercise in humans. J Physiol.

